# Developing a comprehensive database with sensitive health information: A profile of people living with HIV in Newfoundland and Labrador, Canada

**DOI:** 10.23889/ijpds.v5i1.1144

**Published:** 2020-02-25

**Authors:** S Asghari, S Boyd, J Knight, J Blackmore, O Hurley, J Allison, L Gilbert, J Dowden, P Lundrigan

**Affiliations:** 1 Memorial University of Newfoundland Centre for Rural Health Studies, Discipline of Family Medicine, Faculty of Medicine St. John’s, Newfoundland and Labrador, Canada A1B 3V6; 2 Memorial University of Newfoundland Primary Healthcare Research Unit, Discipline of Family Medicine, Faculty of Medicine St. John’s, Newfoundland and Labrador, Canada A1B 3V6; 3 Memorial University of Newfoundland Division of Community Health and Humanities St. John’s, Newfoundland and Labrador, Canada; 4 Eastern Health Newfoundland and Labrador Public Health Microbiology Laboratory St. John’s, Newfoundland and Labrador, Canada A1A 3Z9; 5 Eastern Health Provincial Cancer Care Program St. John’s, Newfoundland and Labrador, Canada A1B 3V6; 6 University of Ottawa Bruyere Research Institute Ottawa, Ontario, Canada K1R 6M1

## Abstract

**Introduction:**

Developing a comprehensive cohort of people living with HIV (PLHIV) to help improve healthcare has long been the vision of researchers, clinicians and decision makers. The development of this kind of database is challenging and requires strict adherence to privacy and confidentiality policies. We explored procedures, activities and events in database development.

**Objectives:**

To understand processes of developing a database with sensitive health information in Newfoundland and Labrador (NL), and to investigate procedures and activities to develop the database within its environmental context.

**Methods:**

A narrative case study was used to explain the challenges and procedures involved in developing a database for our population. The development of the PLHIV cohort in NL is provided as an example to demonstrate the complexity of the process. We linked three datasets that included patient-level data for PLHIV: 1. laboratory data; 2. HIV clinic data; 3. health administrative data, which allowed for the creation of a large database containing many variables describing the PLHIV cohort in the province.

**Results:**

We developed a de-identified cohort of 251 PLHIV that contained 178 variables. Our case study showed database development is an iterative process. The main challenges were ensuring patient privacy and data confidentiality are not compromised and working with multi-custodian data. These challenges were addressed by establishing a data governance team.

**Conclusions:**

It is important that policy be implemented to merge siloed data sources in order to provide researchers with accurate and complete data that is required to conduct sound and precise research with maximum benefits for treatment and policy-making to improve health outcomes.

## Introduction

A comprehensive healthcare database is an effective way to support planning of health services, thus enhancing the delivery of high-quality care [1]. With the continued advancements in technology, the evolution of technology in medicine is inevitable [2,3]. Healthcare researchers are more frequently applying machine learning methods to identify patterns, trends and hidden themes within a patient’s medical record [4-6]. Applying these machine learning methods to health information databases enables the prediction of health risk and early disease prognosis [4-6] which is only made possible with a well-developed, accurate and up-to-date database.

Developing a database that contains sensitive health information, poses many challenges [7]. This is particularly true when collecting data from Indigenous communities, rural communities, underserviced areas, disadvantaged or vulnerable populations or those with rare medical conditions, as identification of an individual is possible based on the data collected [8]. As a result, collecting accurate yet de-identified information about these individuals while meeting ethical research standards may be difficult. However, given the right considerations and approach, a health information database containing sensitive information can be safely developed.

The objective of this study was to understand the processes involved in developing a database with sensitive health information, and to investigate procedures and activities to develop the database within its environmental context. We demonstrated the process using an example of developing a de-identified cohort of PLHIV in NL.

Newfoundland and Labrador (NL) is the easternmost province in Canada with a population of approximately 528,000 and size of 370,514 km2 [9]. The Avalon Peninsula (9220 km2) is home to 270,000 people while the remaining 258,000 are spread out across the remainder of the province with many living in small rural communities [9]. Developing a comprehensive cohort of PLHIV to help improve healthcare has long been the vision of researchers, clinicians and decision makers. Although important to the advancement of healthcare, the creation of this cohort was not without its challenges, which we describe here. In Newfoundland and Labrador, PLHIV primarily receive both specialist and primary care through an interdisciplinary HIV specialty clinic. More recently, some have questioned whether specialists are appropriately addressing the primary care needs of PLHIV [10-13]. Adding family physicians to the continuum of care has the potential to greatly impact the health outcomes and quality of life of PLHIV given the level of care required to effectively manage the other chronic conditions they experience [11, 12]. One of the many objectives of the PLHIV study was to develop a de-identified cohort of PLHIV in NL, to be used to inform policy and prioritize healthcare system changes to optimize HIV healthcare in NL and to address gaps in care for PLHIV. 

## Methods

### Study Design

In this paper, we used a narrative case study approach to explain the challenges and procedures involved in developing a database for our population. We explored procedures, activities and events in database development that include database variables collected using an iterative data extraction technique (including variable identification, database linkage and record de-identification). These procedures and activities took place between January 2013 and December 2017. A list of challenges and steps taken to overcome them were recorded over the course of the study and were subsequently compiled at the end of the study period.

### Data Governance Team

We began by identifying key stakeholders who would be involved in either providing data access, consultation or database development. This included any individual or organizational representative that may influence or be affected by the data pertaining to PLHIV (e.g. AIDS Committee of NL, Provincial HIV Clinic, Memorial University, etc.). Previous studies in the area of database development suggest the involvement of data custodians, information technology experts, database experts and data analysts as key stakeholders [14-16]. In order to obtain insight regarding data applicability and stakeholder expectations, as well as to help resolve issues, we developed a data governance team consisting of individuals from each stakeholder group or organization and included decision makers, clinicians, researchers, database experts, information technology experts, data analysts, learners, and PLHIV. By consulting each of these groups of individuals, all aspects of database development were considered, from obtaining data, to whether patients perceived the development of this database to be beneficial to their health. These team members were kept up-to-date with regard to research progression on a regular basis, were consulted at different stages of database development, and were involved in decision-making.

### Database Development Framework

**Figure 1: Iterative Database Development Process:**
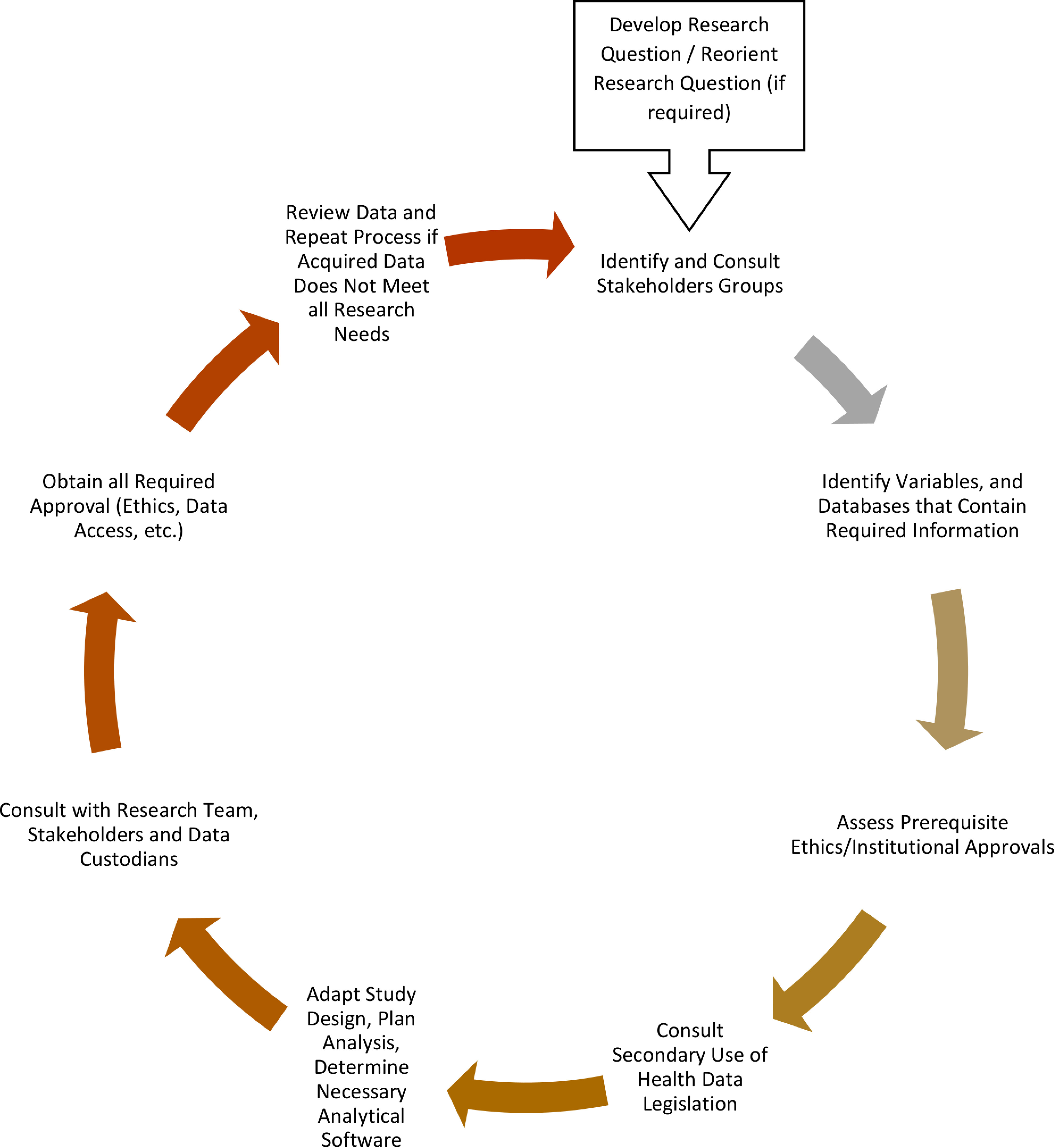
This shows the cycle of an iterative process that repeats itself until research questions and objectives can be answered by the data provided.

Through in-person meetings, we compiled stakeholders’ experiences and expectations related to the development of the HIV database and reviewed their organizational guidelines to create a framework for database development. Given that data in these databases were originally collected for purposes other than research, we met with the data governance team and reviewed available metadata and documentation. These meetings were to familiarize ourselves with the complex structure of the databases, the extent of information contained in available data elements, data interpretation, limitations of the data and possible biases that may have been introduced into the database or associated research activities. We also discussed data policies, use of the datasets for research, the elements of their database approval process and data sharing agreements early in the development process. We developed a list of considerations in using each database and reviewed them with the data governance team to identify possible risk and impact mitigation strategies before data collection and linkage. The framework was iteratively reviewed (See Figure 1) and approved by the data governance team during the data collection process. We regularly met with the data custodians to discuss and resolve any new challenges. These challenges were documented and reviewed to help develop risk and impact mitigation strategies and to determine which parts of the development framework needed revision. In consultation with the data governance team, we also took note of the lessons learned as a result of each challenge encountered that were applicable to future database development.

### Data sources

The data governance team reviewed all organizations/individual researchers who may have collected HIV data in NL. We identified five possible sources of HIV data. Three of these data repositories had the information we needed for our research, which we used for the PLHIV cohort and are described below. Two of the data sources were omitted from the current project. The first of these data sources focused on immunology data rather than health status or healthcare utilization among HIV patients and, thus was not deemed appropriate for the current project. A published paper by Barrett et al provides more detailed information related to the immunology database [17]. The second source (Canadian Primary Sentinel Surveillance Network - CPCSSN) contained electronic medical record primary care data [18, 19]. However, the CPCSSN database contained NL data for only 45,000 patients provided by 51 family physicians, covering less than 10% of the provincial population and less than 10% of family physicians in the province [20] and was not deemed to be ready for data linkage at the time of this study. Included data sources:

1. Newfoundland and Labrador health administrative data and census data.
  The majority of this data is managed by the Newfoundland Labrador Centre for Health Information (NLCHI) and consisted of selected individual-level data elements from provincial health administrative data sources including: the provincial health insurance registry (Medical Care Plan (MCP) Beneficiary Database), MCP physician claims, MCP provider file, hospital discharge abstracts, mortality data, surveillance data from the NL component of the Canadian Chronic Disease Surveillance System and the NL Cancer Registry. Every Canadian province is responsible for providing its residents with free universal healthcare [21]. NL covers the cost of healthcare services for its residents via its provincial Medical Care Plan (MCP) [22]. An MCP number is assigned to every resident of the province and allows healthcare administrators to keep track of every encounter with the healthcare system. Also included in the linked dataset were sociodemographic indicators from the 2006 Statistics Canada Census of Population (e.g. median family income), as well as community-level dependency, deprivation and instability indices from the Canadian Marginalization Index (CMI) produced by McMaster University which utilizes selected data elements from the census. [23]. Before the linked dataset was provided to the research team, census and CMI data were linked to individuals via Statistics Canada’s postal code conversion file, which links six-character postal codes of residence to different levels of aggregation, which in our case was dissemination area level for census data and CMI data. This allowed patients to be classified by approximate census aggregate and health region, and allowed for assignment of small-area level socioeconomic information to patient records without risking identification of an individual through release of individual-level characteristics or lower-level geographic information that has the potential to compromise the privacy of those living with HIV in the province.
2. Public Health Laboratory data
  The provincial Public Health Laboratory of NL provides reference testing, routine and specialized diagnostic screening and infectious disease detection for a multitude of diseases. The lab is the primary site of sexually transmitted blood borne infection diagnostics for the province. This database contains both screening and confirmatory test results for HIV as well as viral load testing at the individual-level and includes individual health insurance numbers.
3. HIV Clinic Data
  The provincial HIV Clinic is located in St. John’s, NL, and provides specialized medical services to PLHIV in the province. The HIV clinic consists of a multidisciplinary team including a physician (infectious disease specialist/internist), nurse practitioner, a social worker and pharmacist who provide care to PLHIV throughout NL, and see each patient approximately every 6-12 months [13]. At the time of this study, the clinic had manual charts including individual-level medication and healthcare information for PLHIV who receive healthcare services via the clinic. The study team entered this data into an electronic database for the purpose of this study using *Microsoft Access*.

The PLHIV database was compiled with the assistance of NLCHI. An individual patient-level identifier in the form of a randomly-generated ID code, which replaced the provincial health insurance number, was used to link the component datasets in order to create the large PLHIV database. The database created from the three data sources contained information regarding each patient’s demographics, laboratory information, hospitalization information, physician visit claims, mortality, physician provider information, cancer related data, comorbidity data, individual risk factor data, pregnancy data, smoking status, community factors (community level socio-demographic data from the Statistics Canada census) and antiretroviral treatment data up to 2015.

### Identifying the study population

Individuals were included in the final database if they met one or more of the following case definitions as determined from component data sources. The accuracy of these case definitions have been reported elsewhere [24, 25].

 1) At least 3 HIV/AIDS diagnostic codes from physician claims (1995-2015), or 1 HIV/AIDS diagnosis code in the hospital discharge abstract (1995-2015) [24].

 OR

 2) A confirmatory HIV test in the Public Health Lab data as described by the government of Canada [25]

 OR

 3) Being included in the HIV clinic patient database indicating that the individual is living with HIV and had received HIV care at the clinic.

### Ethical Considerations, Privacy, and Security

We considered a number of steps and ethical considerations to ensure that individuals in the cohort were not identified, while at the same time maintaining the quality of the data collected. We began by obtaining ethical approval for the use of secondary data from the NL Health Research Ethics Board (HREB). The HREB application included detailed information related to data protection and security, as well as data-sharing strategies and iterative updates. All data extraction and linkage was conducted at NLCHI. NLCHI is a crown corporation responsible for managing provincial health data and information assets and providing health analytics services, whose staff also have expertise in data security and privacy. The final linked dataset was provided to the research team in de-identified form. The data was then transferred to a secure server at Memorial University’s Faculty of Medicine. Only team members conducting the data analysis had access to this server and the security of the server was/is regularly assessed by Memorial University. All team members working with this data were trained in how to work with confidential data and signed an oath of confidentiality.

### De-identification

De-identification is a critical part of working with sensitive data. The NL population has many small communities with a small population and close-knit social networks, which increases the likelihood of individuals with sensitive diseases being identified. NLCHI performed all data linkage and provided the research team with a de-identified linked dataset. De-identification was performed through the removal of health insurance numbers which were replaced by a de-identification key which was created (separately from the current project) through an extensive data quality assurance process, tested and confirmed to be consistent across multiple data sources to allow for accurate data linkage. During de-identification, health insurance numbers are removed from the dataset and replaced with a randomly generated person-level ID number to uniquely identify patients. Potentially identifying variables were either removed from the database or modified to ensure confidentiality (e.g., data contained the first three digits of the postal code (forward sortation area) instead of the full 6-digit postal code).

#### Relevant Legislation

Within NL, there are two significant pieces of legislation that govern the use of personal health information for research:

1) *The Personal Health Information Act* [26] establishes rules that custodians of personal health information must follow when collecting, using and disclosing individuals’ confidential personal health information. The Personal Health Information Act permits custodians to use or disclose personal health information for research purposes without an individual’s consent if a research ethics board under the Health Research Ethics Authority Act approves it.

2) *The Health Research Ethics Act* [27] requires that all health research conducted in the province be reviewed and approved by a local research ethics board in accordance with Tri-council Policy. The Health Research Ethics Authority oversees ethics reviews of health research through the HREB or another approved health research ethics review body. Thus, the inclusion of appropriate data sources for the research project required approval of both the provincial HREB and data custodians. The Personal Health Information Act designates NLCHI, as well as other agencies, as custodians under the Act. The *Centre for Health Information Act* further establishes the authority of NLCHI to collect personal health information from a variety of sources including hospital and mortality data.

### Approvals

As with other research, approval to use administrative and other health-related data is required from several entities before the research can be conducted. Among those required is research ethics board (HREB in NL) approval which requires completion of an application. HREB approval stated that it was the responsibility of the researcher to seek approval from data custodians and other organizations as appropriate. In addition to obtaining HREB approval, a researcher must submit an application to a data custodian to disclose health information. A significant step in the approval process to use secondary health data sources available at NLCHI involves approval from NLCHI’s Secondary Uses Committee. The mandate of the Secondary Uses Committee is to provide guidance on the secondary use of personal health information and review requests for record-level or identifying information by internal and external parties. The Secondary Uses Committee requires an application/privacy impact assessment, variable list and evidence of custodial approval for release or linkage of external data. We also obtained approval for secondary use of data from Eastern Health’s Research Proposal Approval Committee who manage data requests for the provincial HIV clinic and the Public Health Laboratory.

## Results

We found that maximizing the value of secondary data requires a holistic approach before requesting data, during the data acquisition process, as well as during and after analysis. Challenges we encountered during the development of the PLHIV database are listed in table 1. The risk or impact of each challenge are either inherent (i.e. patient privacy and data confidentiality), slow the progress of data acquisition, result in data quality issues, or slow the progress of transforming data to information. The actions taken to overcome these challenges and the subsequent lessons learned can be found in Tables 2a-2d by category.

**Table 1: List of challenges and their potential risk and impact:** 

Potential Risk and Impact	Challenges
Patient privacy & confidentiality	Ensuring patient privacy and confidentiality is not compromised
	Ethics standards compliance

Slow the progress of data acquisition	Data may have multiple custodians
	Processes for requesting data can be cumbersome
	Maintaining data integrity
	Data availability

Data quality	Data consistency
	Data accuracy
	Missing data
	Data comparability

Slow the progress of transforming data to information	Data mining may be complex
	Staff changes
	Optimum data unit (level)
	Choosing an optimal analysis approach

**Table 2a: Action(s) taken and lessons learned for challenges that have a potential risk to patient privacy and confidentiality.** 

Challenges	Action(s) Taken	Lessons Learned
Ensuring patient privacy and confidentiality is not compromised	We minimized the number of data elements requested to only those required to achieve the study’s research objectives. The ethics application also required explanation of the measures taken to ensure patient de-identification. We maintained the data on a secure server to which only the team members conducting the data analyses had access. We avoided citing data in a way that may be identifiable to avoid re-identification, e.g. considering small numbers of patients residing in certain geographic areas.	Consider each variable requested carefully and take necessary measures to minimize the probability of patient re-identification by minimizing the number of data elements requested, safeguarding data access and avoiding data citation in an identifiable manner.

Ethics standards compliance	We reviewed the ethics file regularly to ensure that any changes in the database development were in line with the goals and objectives that were originally approved by the HREB. We regularly assessed that staff, students and researchers who work with data are familiar with the ethics application and implemented ways to ensure the data remained secure. We defined the level of stakeholder access to data/outputs, and data sharing policies to maintain safeguards. We maintained the ethics file throughout the study period, which is a provincially mandated requirement of conducting research that involves the secondary use of health data.	Develop a timeline including electronic reminders for regular review of the ethics files to ensure any changes in the database development is in line with the goals and objectives in the current ethics file. Conduct data access audits as well as review data/information sharing agreements to prevent unauthorized sharing of information with stakeholder groups. Keep the ethics approval active and up to date until the final publication is published.

**Table 2b: Action(s) taken and lessons learned for challenges that slow the progress of data acquisition.** 

Challenges	Action(s) Taken	Lessons Learned
Data may have multiple custodians	We developed a data governance team that included independent researchers and members from provincial data custodians and stakeholder groups. The data governance team provided prerequisite information in order to develop a variable list that would satisfy all research objectives and the repositories that contain the desired health information. We met with each data custodian separately to collect information related to their secondary data request process and to determine which approvals would need to be obtained prior to applying for data access.	Establish a data governance team when required data elements are housed across multiple data custodians. The involvement of data custodians earlier in the database development process is essential.

Processes for requesting data can be cumbersome	The rationale for the inclusion of data elements from different databases and their relation to the study’s research objective was drafted after local stakeholder groups and data custodian representatives were consulted. The rationale included both the preferred method of analysis and the reason each variable was included in our request. Our secondary data request also provided the inclusion dates for each of the variables as well as practical definitions for every variable especially for disease-specific case definitions. Prior to data abstraction and collection, we made sure all ethics-based prerequisites were met. This included taking oaths of confidentiality, obtaining ethics approval from provincial ethics boards and regional health authorities, completing legislated provincial privacy training and receiving custodian-specific approval.	When requesting data, researchers must first be clear on the purpose for creation of the database as well as research objectives. Once the purpose for the data request and research objectives have been established and understood by each of the data custodians, researchers should provide details on how certain health conditions are defined and what software will be used to link and analyze each of the databases.

Maintaining data integrity	To ensure data was not compromised due to human errors or unintended transfer errors, we kept a copy of our original database in a separate folder. The server used to store all related databases was backed‐up on a daily basis to avoid issues related to hardware failure, malware, accidental deletion of critical files or data corruption.	It is important to keep a copy of your original database in a secure location that is routinely backed‐up. Do not assume the data custodian will indefinitely keep a copy of the extracted database.

Data availability	When working with secondary data, there are times when some data is not available for research purposes. We regularly reviewed the approved databases with each of the custodians to determine whether new/up‐to‐date data was available. We discussed the feasibility of adding new databases, updating the database with new years of data over time, and adding data elements with the data custodian, as well as the process of obtaining and linking the new data to the existing database. In some cases, the data was only available in paper‐based records and therefore had to be entered into the database manually using electronic forms that were jointly developed with a provincial data custodian.	Regularly assess the availability of any new data and the feasibility of adding new data to your database. Paper-based record data abstraction requires a significant amount of time and effort and should be rigorously scrutinized to meet the data quality standards required for subsequent data analysis.

**Table 2c: Action(s) taken and lessons learned for challenges that have a potential risk to data quality.** 

Challenges	Action(s) Taken	Lessons Learned

Data consistency	To ensure the data is usable and formatted in a consistent way, we reviewed each variable’s corresponding data to identify an optimum approach when data is transferred between different software (e.g. Excel to Access). We converted the data to the same/similar format when it was applicable to allow for data transfer/linkage. This approach helped to minimize missing information during the data transfer/linkage and helped to avoid issues related to subsequent data analysis. We discussed our preferred data format and preferred statistical software (e.g. SAS, STATA) with data custodians to ensure file/variable type compatibility and to reduce the need for subsequent data conversions.	It is important to ensure that each variable is entered into the database in the intended format and that proper data conversion methods are applied.

Data accuracy	To ensure the data elements included in our database accurately reflected what they were supposed to measure, we developed a protocol to assess the data accuracy throughout the whole process. The protocol included double entry, assessing a random sample of data, reviewing types of data (nominal, ordinal, numeric), and assessing the appropriateness of each variable’s range of data (e.g. age 1001), data units (e.g. age = 15 months). We compared the values for each variable with the expected range, format, unit type, etc. and compared the results with existing national/provincial reports at every stage. We reviewed the data dictionary and learned about the sampling frame, study population and important considerations for secondary data analysis that were brought up by the data custodian. To review the data accuracy of each data source, we compared it to the other databases, and discussed any inaccuracies with the data custodian to identify the best approach to address the inaccuracy.	When working with data, it is important to ensure the data always reflects what it is supposed to measure. To do so, the feasibility of database reviews and revisions at every stage of the study should be considered. This may involve sensitivity analysis through the comparison of the actual database with the expected product, comparing the database to pre-existing databases (if available), and reviewing early outputs with stakeholders. It is also critical to become familiar with sampling frame and the target population of each database. Developing or validating case definitions across multiple sources of data requires some measures on the part of the research team to limit the level of discrepancy between sources.

Missing data	We discussed any variable that contained a large amount of missing data with each of the custodians during the data request process. We planned statistical analysis to deal with missing data through imputation and sensitivity analysis. We interpreted the results of our analysis within the context of the limitations associated with missing data. It is also useful to familiarize yourself with/consult a statistician on approaches for dealing with missing data (e.g. imputation and sensitivity analysis).	Be prepared for missing data when working with secondary data. Reviewing databases with stakeholders is helpful to minimize the impact of missing data. It is also useful to familiarize yourself with/consult a statistician on approaches for dealing with missing data (e.g. imputation and sensitivity analysis).

Data comparability	We evaluated all data elements included in our database to determine whether they conformed to what was originally requested. Content included in databases can change over the course of a study period, which may happen without prior notice. A data dictionary was developed by the research team based on the original data dictionary provided by the data custodian, which contains notes that document the changes made to the original databases during the study period. This more comprehensive data dictionary defined each of the variables (e.g. name, format, description, notes), described missing data, defined acronyms, and provided data ranges, etc. After new analysis was completed, we added information about any changes made to our database (e.g. new variables, composite variables, etc.) and the date of this change to the data dictionary. We regularly reviewed the data dictionary with the research team to ensure it captured all changes made to the originally requested database.	After identifying and obtaining the requested data sources related to the research question, it is very important to develop a data dictionary (metadata) for the purposes of your own record keeping. Data dictionaries should include any changes made by the research team, and an up-to-date list of study variables, and may be different from the one that is provided alongside the original database by the data custodians.

**Table 2d: Action(s) taken and lessons learned for challenges that slow the progress of transforming data to information.** 

Challenges	Action(s) Taken	Lessons Learned
Data mining may be complex	We trained (e.g. pertinent knowledge related to the purpose of the database, how to properly link data elements across sources/tables, etc.) all individuals before being involved in data mining. This minimized the amount of downtime attributed to training/briefing new team members involved in database development. We maintained detailed notes throughout all phases of the database development process to maintain efficiency and to use as reference material if issues are identified later in the study. These notes were useful whenever a new research team member was introduced to the project. We ensured that all individuals on the research team who were involved in the data mining process remained the same for as long as possible.	Studies with relatively long database development periods should try to minimize the number of different staff involved in the project, as much as possible, over time. Keeping a record of the data mining process and detailed notes decreased the impact to the progress and speed of the development process when a change in personnel occurred.

Staff changes	Every new staff member on the research team started with a brief training exercise related to best practices for data mining and data analyses. We developed a document outlining various procedures for staff to review when it was applicable. We had regular meetings with staff so the documents outlining the procedures were kept up-to-date and relevant based on the tasks that needed to be performed. We planned regular training sessions for all staff members to ensure their knowledge related to specific tasks was kept up-to-date.	It is important to have a document outlining procedures related to database development tasks for staff to review when required. Regular training exercises that deal with database development for your staff ensures a standardization of database development methods that should, in turn, improve the overall quality of the end product.

Optimal data unit (level)	Some variables are not available at the desired level requested (e.g. individual or aggregate-level). Once we identified the variables that could not be provided at the level requested, we considered alternative variables/databases that may contain the desired information with data stakeholders and custodians. Once alternatives were fully considered, we planned for subsequent data aggregation and identified the finest level of data available for multi-level data analysis.	Multi-level data analysis (individual vs. group level data) may be required if certain variables are not available at the individual level.

Choosing an optimal analysis approach	Before conducting any analyses, we discussed the key messages and target audiences with our team as well as expected outcomes and knowledge to be disseminated. We reviewed the analytical approach used to address needs that were defined by the research team. We then selected an optimal approach to data analysis and how best to disseminate the results. We reviewed both the format of each variable and the database structure. We broke them down and organized them in a manner that was conducive to answering our research question while also considering the type of expertise required to complete the analysis. We also created simple logics on: How to examine PLHIV data. How to manipulate PLHIV data. How to communicate outputs from PLHIV data analysis.	Throughout all stages of data analysis, you must think about the final product, choose the right platform for your target audience, consider the technology/software required to produce the desired result and consider your expertise and available resources to ensure the analysis is completed in a timely manner and as intended.

**Figure 2: Diagram of the data sources of PLHIV in NL who were still alive at the end of the study period:**
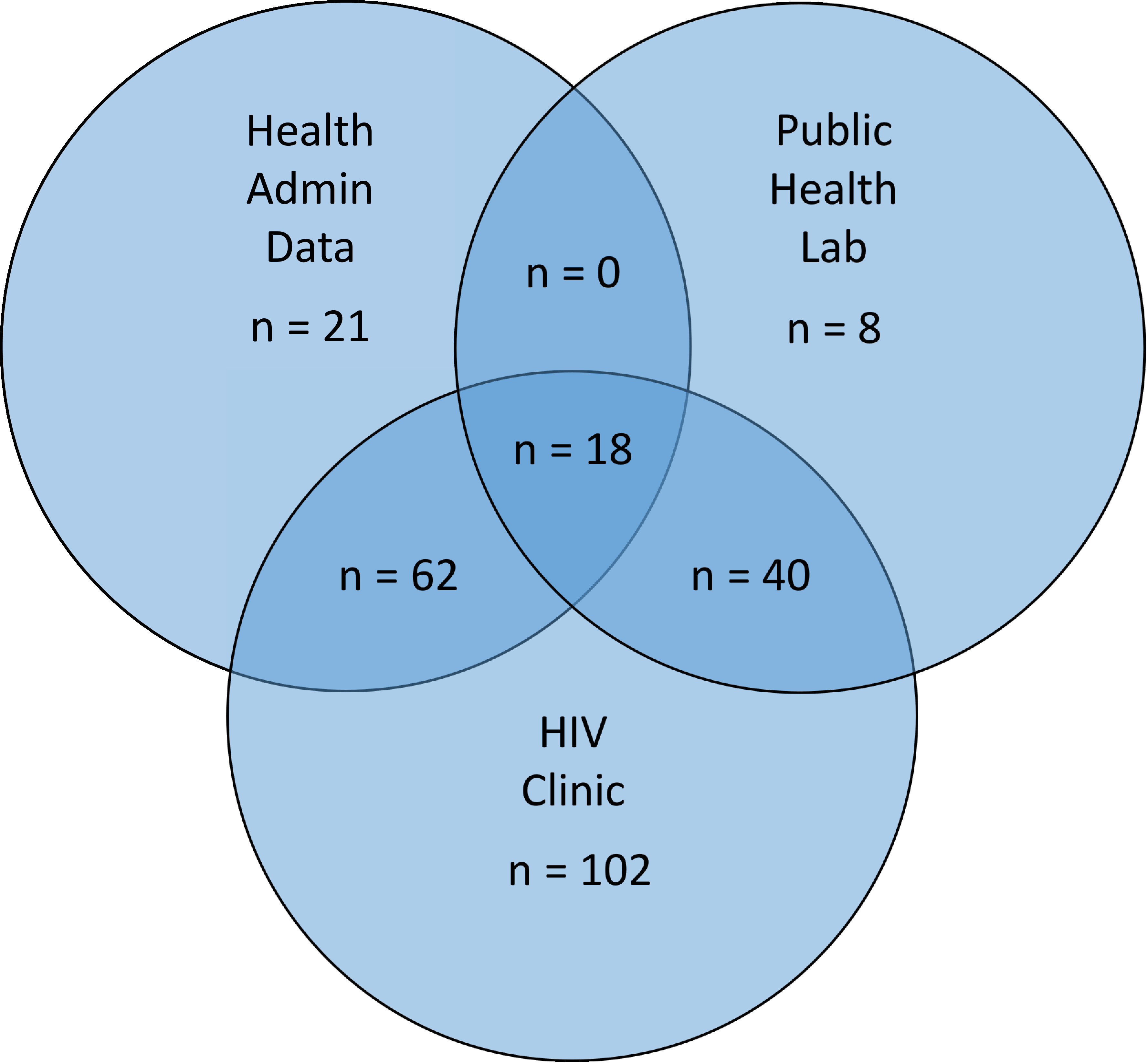


The NL PLHIV cohort database took 3 years to develop due to the challenges described in Tables 2a-2d. The finalized dataset included 317 people who had been diagnosed with HIV in NL. Among these, 66 (20.8%) had died by the end of the study period (December 2015), leaving an active cohort of 251 PLHIV in NL. The final database contained a total of 178 variables describing the health and health care utilization of PLHIV. As shown in Figure 2, 222 (88.4%) of these people were identified through the HIV clinic database, 101 (39%) through health administrative data and 66 (25%) through the Public Health Lab data (not mutually exclusive). Only 18 people (7%) were identified by all three sources and 120 (47.8%) were identified by two data sources.

## Discussion

Comprehensive healthcare databases are important for guiding policy, research and clinical practice and have been shown to be an effective tool to inform delivery of high quality care and improve patient outcomes [1]. We describe several challenges and mitigation strategies that we hope are useful to others seeking to develop similar databases. We also found that not all people with HIV were identified through the multiple sources.

Ideally, all 251 patients in our cohort would have been identified by all three sources; however, only 7% were identified by all three sources. The Public Health Lab has a policy of documenting a positive HIV test for every patient who enters care and requires a viral load test. From this source, however, only 66 patients were identified, which is far lower than the number of known patients in the HIV clinic program. This suggests that a comprehensive database consisting of multiple linked data sources provides a more complete picture for healthcare decision making as opposed to using only a single data source, which may result in missing cases and/or other biases due to a limited population, data inaccuracies or missing information [28-30]. The inconsistencies between data sources was found to be the most difficult challenge to overcome during the creation of the database since the researchers had to hypothesize as to why certain individuals had not been detected by all three sources. As an example, some individuals not identified in the lab data may have been diagnosed before the study period, outside the province, or may have been receiving treatment outside the province. Ideally, all data sets should be linked or somehow integrated/bridged to allow for cross-referencing among multiple data sources.

Another challenge with administrative healthcare datasets is that they traditionally have a lag time of months to years before good quality data becomes available to researchers, meaning data is somewhat outdated and less relevant for policy and research, as conditions may have changed by the time data is released to the research team [31, 32]. Also, additional time was required for data extraction and linkage of the data by analysts at NLCHI. Near real-time data, or at least data available on a daily or even monthly basis, would be more beneficial and relevant for decision-makers and researchers and would reduce data extraction time [33, 34]. In addition to lag-time before data release, there was a lengthy approval process (3 years for this study) in order for researchers to be able to access required data sources. Ideally, there would be a mechanism for researchers to obtain timely access to data in a secure fashion while maintaining confidentiality of the data and ensuring privacy of individuals [35]. NLCHI is currently in the process of working with multiple vendors to enhance the province’s health analytics environment bringing a variety of near real-time data flows from across the province into a secure, robust, consolidated repository. The *Health Data Lab* is a publicly funded NLCHI initiative which allows for timely access to electronic health record data as well as data from more traditional repositories for researchers and health systems users [36].

A limitation of the PLHIV database is that its component data sources came from different data custodians, and thus were in different formats, were collected for different purposes and coverage of these data sources involved different, although overlapping segments of the NL population. The data sources also had different data collection periods and starting points [37, 38]. For example, the provincial health administrative data has been collected and archived electronically for over twenty years while the HIV Clinic at the time of the study was still using paper-based charts, which had to be manually entered into an electronic database in 2014/15 for the purposes of this study (data collection period 1995-2015). Another limitation was that the data was spread across three independent sources. Having to develop a case definition that is different for each data source introduces error and inconsistencies. Another potential limitation involves differences in formatting or poor data quality of identifiers used for linkage such as the health insurance number. In addition, there is often a lack of a standardized approach to linking databases across sources which could be due to poor data quality of identifiers used for linking databases and may result in duplicate cases or other linkage errors [39, 40]. An additional problem with regard to the development of a dynamic cohort is ensuring that the linked database it is kept up-to-date in an efficient and timely manner [41]. Our research team found it difficult to maintain the database given the level of financial and human resources required to keep it up-to-date. The provincial Health Data Lab initiative is well positioned to continue the development of the database produced through this project. Platforms should be put in place to allow for continuous updating of data once original databases have been produced.

We overcame many challenges and developed approaches to working with multiple stakeholders and protecting privacy interests. The database we created is a rich, comprehensive, population-based database including community-level information with longitudinal data over many years linking many health-related data sources that allows for future studies of this population. The challenges encountered during database development will inform refinements to further data linkage projects. 

## Conclusion

We developed a comprehensive healthcare database that is essential to support policy development, enhance delivery of quality care services and improve patient outcomes for PLHIV. To ensure the privacy of the individuals, we restricted access to the individual-level database to the trained members of the research team who conducted the analysis. The database is available for future research projects and summary tables are available upon request; however, those seeking to gain access to the database for additional analysis must first secure approvals from the appropriate authorities. Developing these databases is also the first step toward enhanced analysis using machine learning approaches and developing user-friendly platforms for timely information for decision-making. Using our HIV database as an example, we outlined the challenges often faced by researchers and suggested strategies to overcome issues when developing a database containing sensitive information. It is important that policy be implemented to merge siloed data sources in order to provide researchers and policy-makers with the accurate and complete data that is required for sound research and decision-making. The development of this database is applicable to similar databases for other health conditions. Future studies should investigate how the ongoing linkage of multiple data sources can be approached in NL as well as in other jurisdictions.

## Abbreviations

HREB	Health Research Ethics Board
NL	Newfoundland and Labrador
NLCHI	Newfoundland and Labrador Centre for Health Information
PLHIV	People living with HIV

## References

[1] Higgins TC, Crosson J, Peikes D, McNellis R, Genevro J, Meyers D. Using Health Information Technology to Support Quality Improvement in Primary Care. Rockville, MD: Agency for Healthcare Research and Quality; 2015 Report No.: 15-0031- EF.

[2] Hamet P, Tremblay J. Artificial intelligence in medicine. Metab Clin Exp. 2017;69:S36-40. 10.1016/j.metabol.2017.01.011 28126242

[3] Laal M. Technology in medical science. Procedia-Social and Behavioral Sciences. 2013;81:384-388.

[4] Barak-Corren Y, Castro VM, Javitt S, Hoffnagle AG, Dai Y, Perlis RH, et al Predicting suicidal behavior from longitudinal electronic health records. Am J Psychiatry. 2016;174(2):154-162. 10.1176/appi.ajp.2016.16010077 27609239

[5] Churpek MM, Yuen TC, Winslow C, Meltzer DO, Kattan MW, Edelson DP. Multicenter Comparison of Machine Learning Methods and Conventional Regression for Predicting Clinical Deterioration on the Wards. Crit Care Med. 2 2016;44(2):368-374. 10.1097/CCM.0000000000001571 PMC4736499 26771782

[6] Hung C, Chen W, Lai P, Lin C, Lee C. Comparing deep neural network and other machine learning algorithms for stroke prediction in a large-scale population-based electronic medical claims database. Engineering in Medicine and Biology Society (EMBC), 2017 39th Annual International Conference of the IEEE; IEEE; 2017. 10.1109/EMBC.2017.8037515 29060556

[7] Donaldson MS, Lohr KN. Health Databases and Health Database Organizations: Uses, Benefits, and Concerns. 1994.

[8] Health System Use Technical Advisory Committee Data De-Identification Working Group. 'Best Practice’ Guidelines for Managing the Disclosure of De-Identified Health Information. Ottawa, ON: Canadian Institute for Health Information; 2010.

[9] Statistics Canada. Population and Dwelling Counts [Internet]. 2017 [2019 August 1]. Available from: https://stats.gov.nl.ca/Statistics/Census2016/PDF/Pop_Dwellings_NL_CD_2016.pdf.

[10] Cheng QJ, Engelage EM, Grogan TR, Currier JS, Hoffman RM. Who Provides Primary Care? An Assessment of HIV Patient and Provider Practices and Preferences. J AIDS Clin Res. 11 2014;5(11):10.4172/2155,6113.1000366. 10.4172/2155-6113.1000366 PMC4409003 25914854

[11] Chu C, Selwyn PA. An epidemic in evolution: the need for new models of HIV care in the chronic disease era. Journal of Urban Health. 2011;88(3):556-566. 10.1007/s11524-011-9552-y 21360244 PMC3126936

[12] Kendall CE, Wong J, Taljaard M, Glazier RH, Hogg W, Younger J, et al A cross-sectional, population-based study measuring comorbidity among people living with HIV in Ontario. BMC Public Health. 2014;14(1):161. 10.1186/1471-2458-14-161 PMC3933292 24524286

[13] Kielly J, Kelly DV, Asghari S, Burt K, Biggin J. Patient satisfaction with chronic HIV care provided through an innovative pharmacist/nurse-managed clinic and a multidisciplinary clinic. Canadian Pharmacists Journal/Revue des Pharmaciens du Canada. 2017;150(6):397-406. 10.1177/1715163517734236 29123599 PMC5661679

[14] Xu H. Data quality issues for accounting information systems' implementation: Systems, stakeholders, and organizational factors. Journal of Technology Research. 2009;1:1.

[15] Vayena E, Blasimme A. Health research with big data: Time for systemic oversight. The journal of law, medicine & ethics. 2018;46(1):119-129. 10.1177/1073110518766026 PMC6052857 30034208

[16] Craig T, Ludloff ME. Privacy and big data: the players, regulators, and stakeholders. " O'Reilly Media, Inc."; 2011.

[17] Barrett L, Stapleton SN, Fudge NJ, Grant MD. Immune resilience in HIV-infected individuals seronegative for cytomegalovirus. AIDS. 2014;28(14):2045-2049. 10.1097/qad.0000000000000405 25265072

[18] Canadian Primary Care Sentinel Surveillance Network. Mission & Goals [Internet]. 2016 [2019 August 1]. Available from: https://cpcssn.ca/about-cpcssn/mission-goals/.

[19] Garies S, Birtwhistle R, Drummond N, Queenan J, Williamson T. Data resource profile: national electronic medical record data from the Canadian Primary Care Sentinel Surveillance Network (CPCSSN). Int J Epidemiol. 2017;46(4):1091-1092f. 10.1093/ije/dyw248 28338877

[20] Memorial University of Newfoundland. APBRN/NL-CPCSSN [Internet]. 2019 [2019 August 1]. Available from: https://www.med.mun.ca/phru/apbrn.aspx.

[21] Canada Health Act [Internet]. 2018 [2019 August 1]. Available from: https://www.canada.ca/en/health-canada/services/health-care-system/canada-health-care-system-medicare/canada-health-act.html.

[22] Newfoundland and Labrador health and Community Services. Medical Care Plan (MCP) [Internet]. 2019 [2019 August 1]. Available from: https://www.health.gov.nl.ca/health/mcp/index.html.

[23] Centre for Research on Inner City Health. Canadian Marginalization Index User Guide. Toronto, Ontario: 2012 Report No.: 1 .

[24] Antoniou T, Zagorski B, Loutfy MR, Strike C, Glazier RH. Validation of case-finding algorithms derived from administrative data for identifying adults living with human immunodeficiency virus infection. PloS one. 2011;6(6):e21748. 10.1371/journal.pone.0021748 PMC3128093 21738786

[25] Government of Canada. Human Immunodeficiency Virus - HIV Screening and Testing Guide [Internet]. 2014 [2019 August 1]. Available from: https://www.canada.ca/en/public-health/services/hiv-aids/hiv-screening-testing-guide.html.

[26] Newfoundland and Labrador health and Community Services. The Personal Health Information Act [Internet]. 2019 [2019 August 1]. Available from: https://www.health.gov.nl.ca/health/phia/.

[27] Health Research Ethics Authority Act. Chapter H-1.2 An act to establish a health research ethics authority for the province. In: St. John's, Newfoundland and Labrador, Canada: Queens Printer; 2006.

[28] Van Mourik MS, van Duijn PJ, Moons KG, Bonten MJ, Lee GM. Accuracy of administrative data for surveillance of healthcare-associated infections: a systematic review. BMJ open. 2015;5(8):e008424 10.1136/bmjopen-2015-008424 PMC4554897 26316651

[29] Roos LL, Walld R, Wajda A, Bond R, Hartford K. Record linkage strategies, outpatient procedures, and administrative data. Med Care. 1996:570-582. 10.1097/00005650-199606000-00007 8656723

[30] Holman CDJ, Bass JA, Rosman DL, Smith MB, Semmens JB, Glasson EJ, et al A decade of data linkage in Western Australia: strategic design, applications and benefits of the WA data linkage system. Australian Health Review. 2008;32(4):766-777. 10.1071/AH080766 18980573

[31] Evan WM. Organizational lag. Hum Organ. ;():-1966;25(1):51-53.

[32] Cottle M, Hoover W, Kanwal S, Kohn M, Strome T, Treister N. Transforming Health Care Through Big Data Strategies for leveraging big data in the health care industry. Institute for Health Technology Transformation, http://ihealthtran.com/big-data-in-healthcare. 2013.

[33] Lavis JN. Research, public policymaking, and knowledge‐translation processes: Canadian efforts to build bridges. J Contin Educ Health Prof. 2006;26(1):37-45. 10.1002/chp.49 16557509

[34] Pampel H, Vierkant P, Scholze F, Bertelmann R, Kindling M, Klump J, et al Making research data repositories visible: The re3data.org registry. PloS one. 2013;8(11):e78080. 10.1371/journal.pone.0078080 PMC3817176 24223762

[35] Mcgrail KM, Davidson H, Gavin F, Diverty B, Ethier J-, Katz A, et al SPOR National Data Platform. 2019; Available at: https://www.canada.ca/en/institutes-health-research/news/2019/04/minister-of-health-announces-81m-initiative-to-increase-access-to-health-research-data.html. Accessed July/28, 2019.36.

[36] Zeidenberg J. Newfoundland jump-starts its work in health analytics. Canadian Healthcare Technology. 2019.

[37] Asghari S, Mahdavian M. Secondary analysis of electronic databases: potentials and limitations. Diabetologia. 2013;56(9):2096-2097. 10.1007/s00125-013-2979-9 23811811

[38] Botsis T, Hartvigsen G, Chen F, Weng C. Secondary use of EHR: data quality issues and informatics opportunities. Summit on Translational Bioinformatics 2010;2010:1. PMC3041534 21347133

[39] Haddad N, Li JS, Totten S, McGuire M. HIV in Canada—Surveillance Report, 2017. Can Commun Dis Rep; 2018 Report No.: 44(12):324-332. 10.14745/ccdr.v44i12a0330906230

[40] Government of Canada. HIV and AIDS in Canada: Surveillance Report to December 31, 2014 [Internet]. 2015 [2019 August 1]. Available from: https://www.canada.ca/en/public-health/services/publications/diseases-conditions/hiv-aids-canada-surveillance-report-december-31-2014.html.

[41] Shoemaker ES, Becker ML, Liddy CE, McClarty LM, Asghari S, Hurd J, Rourke SB, Shaw SY, Bibeau C, Rosenes R, Lundrigan P, Crowe L, Ireland L, Loeppky C, Kendall CE. (2019). Creating clinical cohorts: Challenges encountered in two Canadian provinces. Healthcare Policy. 15(1): 10-19. 10.12927/hcpol.2019.25942 PMC7008696 31629452

